# *Ex vivo* assessment of sulbactam-durlobactam clearance during continuous renal replacement therapy to guide dosing recommendations

**DOI:** 10.1128/aac.01674-23

**Published:** 2024-12-10

**Authors:** Yasmeen Abouelhassan, Yuwei Shen, April Chen, Xiaoyi Ye, David P. Nicolau, Joseph L. Kuti

**Affiliations:** 1 Center for Anti-Infective Research and Development, Hartford Hospital 23893 , Hartford, Connecticut, USA; 2 Innoviva Specialty Therapeutics Inc 706261 , Waltham, Massachusetts, USA; 3 Division of Nephrology, Hartford Hospital 23893 , Hartford, Connecticut, USA; 4 Division of Infectious Diseases, Hartford Hospital 23893 , Hartford, Connecticut, USA; Providence Portland Medical Center, Portland, Oregon, USA

**Keywords:** sulbactam-durlobactam, *Acinetobacter baumannii*, renal replacement, CVVH, dosing, pharmacodynamics, Monte Carlo simulation

## Abstract

Sulbactam-durlobactam is approved for the treatment of hospital-acquired and ventilator-associated bacterial pneumonia caused by susceptible isolates of *Acinetobacter baumannii-calcoaceticus* complex. Patients with serious *Acinetobacter* infections may require support with continuous renal replacement therapy (CRRT), which presents challenges for optimal dosing of antibiotics. Sulbactam-durlobactam dosing regimens were derived for this population using an *ex vivo* CRRT model and Monte Carlo simulation (MCS). Transmembrane clearance (CL_TM_) was determined in hemofiltration (CVVH) and hemodialysis (CVVHD) modes using the Prismaflex M100 and HF1400 hemofilter sets and with effluent rates of 1, 2, and 3 L/h. Pre-filter, post-filter blood, and effluent samples were collected over 60 min to calculate sieving (SC) and saturation (SA) coefficients for CVVH and CVVHD, respectively. An established population pharmacokinetic model was integrated with the CL_TM_; then, a 1,000 patient MCS was conducted to determine exposures of potential dosing regimens. Adsorption and degradation in the *ex vivo* CRRT model were negligible. The overall mean ± standard deviation SC/SA was 1.14 ± 0.12 and 0.93 ± 0.08 for sulbactam and durlobactam, respectively. In multivariable regression analyses, effluent rate was the primary driver of CL_TM_ for both drugs. For effluent rates <3 L/h, sulbactam-durlobactam 1 g-1g q8h as 3 h infusion achieved a high probability of pharmacodynamic target attainment while retaining area under the curve exposures consistent with the standard dose in non-CRRT patients. For effluent rates ≥3 to 5 L/h, the optimal regimen was 1 g-1g q6h 3 h infusion. Sulbactam-durlobactam regimens that provide optimum drug exposures for efficacy and safety were identified for CRRT based on the prescribed effluent rate.

## INTRODUCTION

The global emergence of multidrug-resistant *Acinetobacter baumannii* and related species is an area of great concern. In its 2019 antibiotic-resistance threat report, carbapenem-resistant *A. baumannii* (CRAB) was labeled an urgent threat by the Centers for Disease Control and Prevention (CDC) (1). It is a common cause of hospital-acquired bacterial pneumonia (HABP), ventilator-associated bacterial pneumonia (VABP), and bacteremia (2, 3). Currently, few antimicrobials remain active against CRAB (4). Sulbactam (SUL), traditionally used as a class A β-lactamase inhibitor, has intrinsic activity against *A. baumannii* and remains a first line therapy for infections due to this pathogen (5). However, SUL can be inactivated by certain β-lactamases produced by *A. baumannii*, including class D oxacilllinases. Durlobactam (DUR) is a novel diazabicyclooctane (DBO) β-lactamase inhibitor that exhibits potent inhibition of class A, C, and D enzymes and, therefore, restores sulbactam susceptibly to *A. baumannii* strains that produce these β-lactamases (6). Sulbactam-durlobactam (SUL-DUR) (Xacduro, Innoviva Specialty Therapeutics, La Jolla, CA) is approved by the Food and Drug Administration for the treatment of HABP and VABP caused by susceptible isolates of *Acinetobacter baumannii-calcoaceticus* complex (7). In the randomized, Phase 3, clinical trial, SUL-DUR was non-inferior to colistin for 28-day all-cause mortality, while resulting in superior clinical response and safety (8). The approved dose for patients with normal kidney function (i.e., creatinine clearance, CL_cr_ of 45–129 mL/min) is 1 g of SUL and 1 g of DUR (1 g-1g) q6h administered as a 3 h infusion (7). Both SUL and DUR are primarily eliminated by glomerular filtration, with SUL also undergoing active secretion through organic anion transporter 1 (OAT1). As a result, dosage modifications are recommended for patients with moderate to severe renal dysfunction, including those requiring intermittent hemodialysis.

Many patients with HABP or VABP caused by *A. baumannii* may also develop acute kidney injury and require continuous renal replacement therapy (CRRT) for support, including continuous veno-venous hemofiltration (CVVH), continuous veno-venous hemodialysis (CVVHD), and continuous veno-venous hemodiafiltration (CVVHDF) (9, 10). CRRT is progressively replacing intermittent hemodialysis in critically ill patients with severe acute kidney injury, and the use of high-flow dialysis filters combined with aggressive treatment flow rates may result in considerable drug removal (9–11). The altered clearance during CRRT is reported to cause suboptimal antibiotic exposures and may lead to therapeutic failure (12–14).

Clinical data to support dosing recommendations during CRRT are often scarce and not sufficient to make dosing recommendation based on different CRRT modalities, hemofilter types, and effluent flow rates. In the absence of clinical data, *ex vivo* CRRT models can provide valuable information to estimate the sieving coefficient (SC for CVVH) and saturation coefficient (SA for CVVHD) and, thus, calculate the transmembrane clearance (CL_TM_) for various filters and effluent flow rates (15–17). When CL_TM_ is added to a drug’s non-renal clearance (CL_NR_), a reasonable estimate of total clearance for the drug can be employed during simulation to determine optimal dosage regimens that mimic the area under the curve (AUC) and free time above the MIC (%*f*T >MIC) exposures of patients with normal kidney function. Herein, we sought to characterize SUL and DUR adsorption, protein binding, and CL_TM_ in an *ex vivo* CRRT model using bovine blood and then integrate the derived CL_TM_ with established SUL-DUR population pharmacokinetic models to propose dosing regimens for patients on CRRT.

## RESULTS

### Degradation and adsorption studies

Neither SUL nor DUR displayed degradation during the 60 min experiment. The urea dilution factor at 60 min across all adsorption experiments was 68.2 ± 1.5. Minimal adsorption to the hemofilter sets were observed for SUL (values ranged from 7%–11% across experiments) and DUR (8%–12%). The adsorption was 1%–5% higher in the CVVHD mode compared with CVVH mode for the same filter (Table S1) and peaked at 10–20 min before declining. Due to the lack of degradation and minimal adsorption in the *ex vivo* experiments, these parameters were not incorporated into the dosing simulations.

### Protein binding

SUL protein binding in bovine plasma at 0 and 60 min was 0.7% ± 7.9% and 0.0% ± 5.9%, respectively. DUR protein binding was 16.5% ± 5.5% and 19.8% ±3.9% at 0 and 60 min, respectively.

### CRRT clearance studies

**Fig 1:**
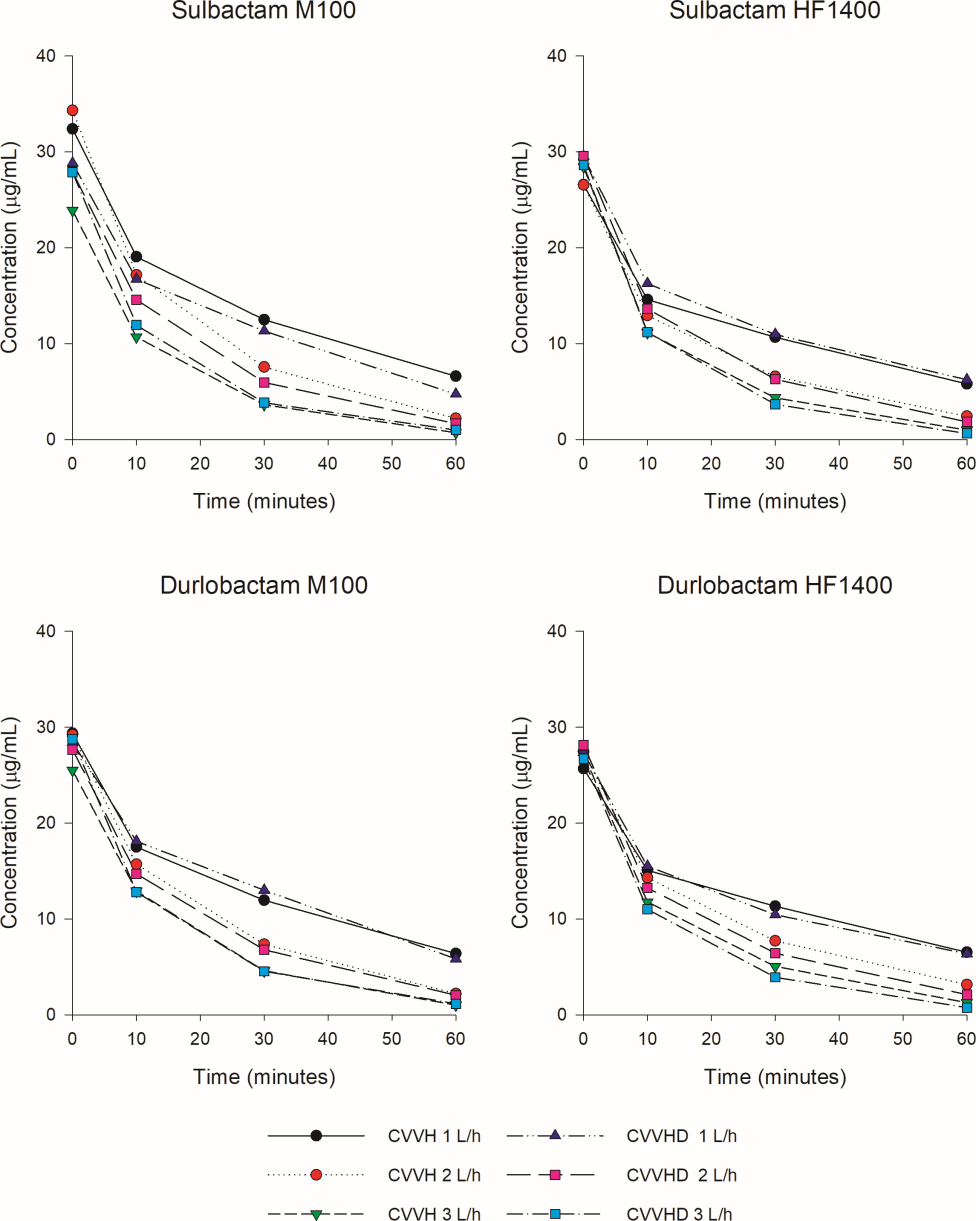
Observed plasma concentrations of sulbactam and durlobactam over time in the *ex vivo* CRRT central reservoir by CRRT mode and filter type.

The mean ± standard deviation (SD) starting concentrations of SUL and DUR in the central reservoir of the *ex vivo* model were 29.0 ± 3.0 and 28.2 ± 3.3 µg/mL across all experiments, respectively, which corresponds with approximate peak concentrations for both drugs in humans (7). Concentrations declined over the 60 min experiments, and the rate of decline increased proportionally with increasing effluent flow rates (Fig. 1). Mean ± SD of the SC and SA values for SUL and DUR according to the CRRT mode, filter type, effluent flow rate, and point of replacement fluid dilution are reported in Table 1. Both drugs were efficiently cleared during CVVH and CVVHD using either hemofilter set (i.e., M100 or HF1400) with mean SC/SA values of 1.14 ± 0.12 and 0.93 ± 0.08 for SUL and DUR, respectively. For SUL, SC/SA was significantly greater in CVVHD mode (1.11 ± 0.12 vs 1.22 ± 0.09, *P* = 0.001), through HF1400 hemofilters (1.10 ± 0.11 vs 1.18 ± 1.12, *P* = 0.007), and with 100/0% dilution (1.13 ± 0.11 vs 0.93 ± 0.09, *P* < 0.001). For DUR, there were no significant differences by mode (CVVH: 0.91 ± 0.07 vs CVVHD: 0.95 ± 0.10, 9 = 0.061), hemofilter (M100: 0.92 ± 0.08 vs HF1400: 0.94 ± 0.09, *P* = 0.236), and point of dilution (100/0%: 0.92 ± 0.06 vs 50/50%: 0.85 ± 0.06, *P* = 0.126).

**TABLE 1:** Sieving (SC) and saturation coefficient (SA) of SUL and DUR during the *ex vivo* CRRT clearance runs by mode, effluent rate, replacement fluid, and filter type^*c*^

CRRT mode, pre-/post-replacement	SUL		DUR	
CVVH	M100	HF1400	M100	HF1400
	SC	SC	SC	SC
1 L/h, 100/0%^*a*^	1.07 ± 0.08	1.19 ± 0.08	0.94 ± 0.06	0.91 ± 0.03
2 L/h, 100/0%^*a*^	1.00 ± 0.08	1.19 ± 0.09	0.91 ± 0.11	0.92 ± 0.05
2 L/h, 50/50%^*b*^	1.00 ± 0.01	0.84 ± 0.02	0.89 ± 0.03	0.83 ± 0.06
3 L/h, 100/0%^*a*^	1.10 ± 0.07	1.19 ± 0.08	0.93 ± 0.04	0.92 ± 0.09
CVVHD	SA	SA	SA	SA
1 L/h	1.23 ± 0.06	1.17 ± 0.03	0.93 ± 0.10	0.97 ± 0.07
2 L/h	1.27 ± 0.06	1.23 ± 0.09	0.94 ± 0.17	1.07 ± 0.11
3 L/h	1.10 ± 0.07	1.29 ± 0.13	0.81 ± 0.09	1.02 ± 0.08

^*a*^ 100/0% pre-replacement fluid included a combination of studies using pre-blood pump (PBP) rates of 800–2,900 mL/h, pre-replacement flow rates of 0 or 100 mL/h 0.9% sodium chloride for injection, and post replacement fluid of 100 or 200 mL/h 0.9% sodium chloride for injection. These combined rates resulted in pre-/post-replacements of 80%/20% to 97%/3%, which are collectively termed 100%/0% in Table 1 and all statistical analyses on transmembrane clearance.

^*b*^ 50%/50% pre-replacement fluid was 1,000 mL/h of PBP combined with 1,000 mL/h of post-replacement fluid. These studies were only conducted at a total effluent rate of 2 L/h.

^*c*^ Values are mean ± SD of triplicate runs. SUL, sulbactam; DUR, durlobactam; CVVH, continuous veno-venous hemofiltration; CVVHD, continuous veno-venous hemodialysis.

Multiple linear regression was performed to evaluate whether the filter type, the CRRT mode, replacement fluid point of dilution, the effluent flow rate, or a combination of these parameters would influence SUL and DUR CL_TM_. For SUL, effluent rate, CRRT mode, and filter were significant predictors of CL_TM_ (Table 2). Additional multivariate linear regression on just the CVVH runs was conducted to further assess the impact of replacement fluid as this variable displayed multicollinearity with the CRRT mode (Table S2); in these analyses, replacement fluid became a significant variable associated with SUL CL_TM_ but the slope coefficient values remained similar to the full model. Notably, the slope coefficient for effluent rate in both models was numerically greater than coefficients for the other significant parameters, and running a linear regression with just effluent rate provided very similar coefficient terms. To simplify CL_TM_ predictions for the Monte Carlo simulations, the final linear regression was reduced to only include effluent flow rate: SUL CL_TM_ (L/h) = 0.114 + (0.973 × effluent flow rate, L/h); (adjusted *R*^2^ = 0.831, *P* < 0.001, Fig. S1a). A comparison of SUL CL_TM_ values for the effluent flow rate only model vs various hemofilter and mode scenarios from the full model is provided in Table S3.

**TABLE 2:** Multiple linear regression analyses of the effect of filter type, CRRT mode, replacement fluid, and effluent flow rate on SUL and DUR CL_TM_^*c*^

	Coefficient	Std. error	*P*-value
SUL (adjusted *R*^2^ = 0.928)			
Constant	0.185	0.258	0.477
Filter type (1 = M100, 0 = HF1400)	−0.169	0.059	0.007
CRRT mode (1 = CVVH, 0 = CVVHD)	−0.324	0.146	0.031
Replacement fluid^*a*^	0.191	0.119	0.116
Effluent flow rate^*b*^	0.973	0.038	<0.001
DUR (adjusted *R*^2^ = 0.898)			
Constant	0.439	0.24	0.073
Filter type (1 = M100, 0 = HF1400)	−0.043	0.055	0.444
CRRT mode (1 = CVVH, 0 = CVVHD)	−0.380	0.136	0.007
Replacement fluid^*a*^	−0.017	0.111	0.880
Effluent flow rate^*b*^	0.760	0.035	<0.001

^*a*^ 1 = 100%/0% pre-replacement, 0 = 50%/50% pre-replacement.

^*b*^ Effluent flow rates were assessed as continuous values in L/h.

^*c*^ SUL, sulbactam; DUR, durlobactam; Std. Error, standard error; CRRT, continuous renal replacement.

For DUR, effluent rate and CRRT mode were significantly predictors of CL_TM_ (Table 2); furthermore, replacement fluid did not impact DUR CL_TM_ in the CVVH only analyses (Table S2). Similar with SUL, the coefficient for effluent flow rate provided the greatest weight to the CLTM estimate; therefore, allowing a simplified regression to be conducted with only effluent flow rate: DUR CL_TM_ (L/h) =0.134 + (0.760 × effluent flow rate, L/h); (adjusted *R*^2^ = 0.835, *P* < 0.001, Fig. S1b). A comparison of DUR CL_TM_ values for the effluent flow rate only model vs various mode scenarios from the full model is provided in Table S4.

### Dose optimization

Monte Carlo simulation for VABP patients with CL_CR_ of 30–129 mL/min receiving the standard 1 g-1g q6h, 3 h infusion dosing regimen resulted in a mean SUL AUC_24-48_ of 466 mg*h/L (+/− one SD range: 357–575), which matched the mean AUCs reported for the 162 participants from clinical trials across the first three days (AUC day 1, 466 mg*h/L; AUC day 2, 517 mg*h/L; AUC day 3, 472 mg*h/L, data on file, Entasis Therapeutics). The mean DUR AUC_24–48_ from the Monte Carlo simulation was 452 mg*h/L (+/− one SD range; 384–520), again matching the 162 patients AUC of 456 mg*h/L (day 1), 504 mg*h/L (day 2), and 451 mg*h/L (day 3). The AUCs, CV, 25th-percentile, and 75th-percentile for the simulated VABP patients are reported in Tables S5 and S6. These simulations set the AUC boundaries used for the CRRT simulations.

Monte Carlo simulations at effluent flow rates of 1–5 L/h in 1 L/h increments were used to predict drug concentrations following various dosing regimens, and the probability of target attainment (PTA) and AUC_24–48_ were calculated. The PTA and AUC_24–48_ simulation results for each dosing regimen at each effluent flow rate is provided in Tables S5 and S6 for SUL and DUR, respectively. All simulated SUL and DUR dosing regimens resulted in PTA > 0.9 for achieving their pharmacodynamic thresholds at 4/4 µg/mL. AUC_24–48_ were overall greater for DUR compared with SUL. Based on a priori criteria (see Materials and Methods), two optimal dosing regimens were selected over the range of simulated effluent flow rates to allow simplification. The proposed regimens were SUL-DUR 1 g-1g q8h as a 3 h infusion for effluent flow rates 1 to <3 L/h and SUL-DUR 1 g-1g q6h as a 3 h infusion for effluent flow rates ≥3–5 L/h. Sensitivity analyses conducted on various CRRT mode and hemofilter scenarios resulted in similar exposures at 2 and 3 L/h effluent rates, thus supporting the proposed dosing regimens (Tables S7 and S8).

## DISCUSSION

This study determined SUL-DUR CL_TM_ in CVVH and CVVHD CRRT modalities across various effluent flow rates, point of replacement fluid dilutions, and two widely used hemofilters, including the propensity for each compound to adsorb to the CRRT circuit. Neither SUL nor DUR demonstrated appreciable adsorption to the M100 and HF1400 hemofilter sets, which is consistent with other β-lactams (15–17). Both SUL and DUR demonstrated high SC and SA values that suggest they are freely cleared through these hemofilters. Using the CL_TM_ derived from the *ex vivo* model and in combination with estimates for non-renal clearance and population pharmacokinetic parameter estimates from patients sampled during the SUL-DUR clinical trials, dosing regimens optimized to both pharmacodynamic exposure and safety (based on AUC) were identified at each effluent flow rate. These data can provide preliminary guidance to optimal SUL-DUR dosing in patients receiving CRRT modalities.

The primary variable affecting the CL_TM_ of both drugs was the effluent flow rate. CRRT mode, hemofilter, and replacement fluid (CVVH only) were significant variables in the SUL multilinear regression model, but their contributions to CL_TM_ were minor and would not likely impact dosage selection (Tables S3 and S7). A similar observation was made for DUR, with CRRT mode significantly contributing to CL_TM_, but not resulting in a sufficient impact to arrive at a different DUR dosage (Tables S5 and S8). These observations are similar to prior CRRT studies with other β-lactams (15–17). Therefore, two dosing regimens were selected based only on the effluent flow rate with no changes required for hemofilter type, CRRT mode, or point of replacement fluid dilution. A dose of SUL-DUR 1 g-1g q8h with each dose administered as a 3 h infusion provided a similar AUC to a standard dosage in VABP patients as per the prescribing instructions and a high PTA at relevant MICs for both drugs for effluent flow rates <3 L/h. Likewise, the standard 1 g-1g q6h (3 h infusion) regimen provided similar AUCs and optimal PTA for effluent flow rates of 3–5 L/h.

Protein binding is a vital attribute that can impact the ability of a drug to pass through contemporary hemofilters. During the *ex vivo* studies, minimal protein binding in bovine plasma was observed for SUL, whereas protein binding in healthy humans is reported to be 38% (7). This species difference in protein binding could impact estimates of SC and SA with CL_TM_ values based on the bovine plasma greater than potentially observed in patients. It is not uncommon for critically ill patients supported on CRRT to have hypoalbuminemia. As a result, the dosage regimens selected were done so to aggressively achieve optimal pharmacodynamic exposure should CL_TM_ in patients be similar to predicted by the ex vivo models. We felt this was a reasonable compromise considering the safety of SUL-DUR in patients. In contrast, DUR protein binding was largely similar to that observed in humans (~18% vs 10% in humans) (7).

The goal AUC values were chosen arbitrarily by selecting a dose for each compound that resulted in a mean exposure during the Monte Carlo simulation that was within one standard deviation from the mean AUC in patients in Phase 2 and 3 who received the full approved dose of 1 g-1g q6h (3 h infusion) and who had a CL_CR_ of 30–129 mL/min. Notably, these ranges were similar to the 25th–75th percentiles of the population (Tables S5 and S6). Due to the complexity of considering two drugs with slightly different pharmacokinetics and CL_TM_, SUL exposure was prioritized over DUR because it is the active drug component of the combination. Fortunately, at the same dose, DUR exposures remained higher compared with SUL because of its lower CL_TM_; therefore, DUR AUC exposures for the recommended dosing regimens were on the higher range of those observed during Phase 2 and Phase 3 clinical trials. However, the excellent safety and tolerability of the compound during registrational trials were taken into consideration to support the higher dosage recommendation.

This study is not without limitations. While multiple CRRT modes, common hemofilter types, and typical effluent flow rates were included, the observations might not apply to other hemofilters circuits made from other materials or other CRRT systems. Adsorption studies were only done at a single effluent flow rate but were minimal and we hypothesize would only decrease at higher flow rates. Furthermore, 100% post-replacement fluid was not evaluated in this study since this method (although efficient at solute removal) is not commonly prescribed due to higher rates of hemofilter clotting. Second, residual renal function was not accounted for due to the *ex vivo* nature of the model. Additional residual urine output in critically ill patients supported by CRRT could result in lower drug exposures as kidney function improves; however, the regimens selected provide sufficient PTA even at effluent rates higher than those proposed and should, therefore, be reasonable estimates. The non-renal clearances and dispersion used during Monte Carlo simulation for both drugs were estimates from patients with chronic renal failure, and the non-renal clearances in critically ill patients with acute kidney injury are unknown. In this study, the CL_TM_ from CVVH and CVVHD were determined separately, while many patients in the ICU receive both filtration and dialysis (i.e., CVVHDF). Although not directly measured, CL_TM_ for CVVHDF should be additive and the overall effluent dosage can be determined by the sum of the pre-filter replacement fluid rate (L/h), the post-filter replacement fluid rate (L/h), the dialysate rate (L/h), the fluid removal rate (L/h), and the pre-blood pump fluid rate (L/h). Finally, no pharmacokinetic data from patients receiving CRRT with known filter types and effluent dosages are currently available to validate the exposures of the proposed dosing regimens. A clinical pharmacokinetic study to evaluate these dosing regimens, as well as any effect of residual urine output, is warranted.

In summary, this study observed SUL and DUR to be efficiently cleared by both CVVH and CVVHD through M100 and HF1400 hemofilters. The clearance of both drugs during CRRT was dependent primarily on the effluent flow rate. When CL_TM_ is incorporated into established population pharmacokinetic models, SUL-DUR dosing regimens of 1 g-1g q8h and q6h (as 3 h infusions) for effluent flow rates of 1 to <3 L/h and ≥3 to 5 L/h, respectively, would provide high pharmacodynamic target attainment against susceptible *Acinetobacter baumannii* (MICs ≤ 4/4 µg/mL) and similar AUC exposures to the standard dosing in VABP patients with CL_CR_ of 30–129 mL/min.

## MATERIALS AND METHODS

### *Ex vivo* CRRT model

*Ex vivo* CRRT was simulated using a Prismaflex 7.2 control unit (Baxter Healthcare Corporation, Deerfield, IL, USA) in CVVH and CVVHD modes. Two hemofilters were tested: 1.4 m^2^ polyarylethersulfone (PAES; Prismaflex HF1400 set; Baxter Healthcare Corporation, REF #106697) and 0.9 m^2^ acrylonitrile (AN69; Prismaflex M100 set; Baxter Healthcare Corporation, REF #107142). At the beginning of each experiment, the Prismaflex circuit was primed with 0.9% sodium chloride per the manufacturer’s instructions. For each run, one liter of heparinized bovine blood (20 units/mL; Lampire Biological Laboratories, Pipersville, PA, USA; blood was stored at 4°C for up to 21 days; hematocrit, 38.7% ± 1.8%; albumin, 3.6 ± 0.1 g/dL) was stirred continuously in a 2 L beaker (i.e., central reservoir) stored in a water bath at 37°C and allowed to circulate throughout the system for 10 min to permit equilibration prior to adding SUL and DUR. Throughout the runs, the blood flow rate was fixed at 200 mL/min, which is consistent with rates used clinically. CVVH replacement fluid (PrismaSOL BGK 2/0; Baxter Healthcare Corporation) and CVVHD dialysate (PrismaSATE BGK 2/0; Baxter Healthcare Corporation) flow rates of 1, 2, and 3 L/h were tested with each filter type. During CVVH at all rates, pre-blood pump (PBP) rates were set between 0.8–2.9 L/h with pre-replacement flow settings of 0 or 100 mL/h 0.9% sodium chloride for injection, or post replacement fluid of 100 or 200 mL/h 0.9% sodium chloride for injection; this is consistent with CRRT prescriptions at our hospital and resulted in pre-/post-replacements of 80%/20% to 97/3%, which are collectively termed 100%/0% in all analyses. To evaluate 50/50 replacement fluid in CVVH mode, the PBP was set to 1 L/h combined with 1 L/h of post-replacement fluid. The 50/50 studies were only conducted at a total effluent rate of 2 L/h. Patient fluid removal was fixed at zero for all runs. All experiments were performed in triplicate in each mode, at each rate, and with each filter.

### Study drug preparation and administration

SUL sodium 1 g/vial (Entasis Therapeutics, lot 2011184) was reconstituted with 5 mL sterile water for injection. DUR (EXT2514) 500 mg/vial (Entasis Therapeutics, lot B21110127) was reconstituted with 2.5 mL sterile water for injection. Once reconstituted, both drugs were separately diluted in 0.9% NaCl to a concentration of 5 mg/mL and stored at −80°C until use. The drug stocks were allowed to thaw on ice for 1 h prior to the study. The drugs were then injected into the central reservoir to reach an approximate total plasma target concentration of 30 µg/mL, which is the total peak concentration observed in healthy subjects administered SUL-DUR 1 g/1 g q6h as a 3 h infusion. Following drug injection, the system was allowed to equilibrate for 1 min prior to sampling. Following equilibration, serial sampling from the central reservoir started immediately after adding the drug (initial concentration, 0 min^−2^), after 2 min equilibration (0 min), and at 10, 30, and 60 min following equilibration. Pre-filter blood samples, post-filter blood samples, and effluent samples were collected at 10, 30, and 60 min from the circuit ports. All blood samples were collected in K_2_EDTA vacutainer tubes (Becton Dickinson, Franklin Lakes, NJ, USA). Blood tubes were centrifuged at 2,000× gravity for 15 min at 4°C, and the plasma was aliquoted into TrueNorth cryovials (Millipore Sigma, Rockville, MD, USA). To ensure the stability of DUR in plasma, all pre- and post-filter plasma samples were diluted 1:1 with a suspension of Pierce Protease Inhibitor Mini Tablets (Product# A32953, ThermoScientific, Waltham, MA, USA). The protease inhibitor suspension was prepared by dissolving one tablet in 10 mL of deionized water and stored at 2–8°C for 2 weeks. The effluent samples were collected in TrueNorth cryovials (Millipore Sigma, Rockville, MD, USA) and did not require the addition of the protease inhibitor. All plasma and effluent samples were frozen at −80°C within 30 min of collection until bioanalytical analysis. The methodology for determining protein binding for SUL and DUR in bovine plasma during all experiments is provided in the supplemental material.

### Degradation studies

To account for any degradation of SUL or DUR in the bovine whole blood over the 60 min experiment, a control model was tested in triplicate. The control model was a 300 mL glass chemostat jar filled with 100 mL of bovine whole blood (i.e., the same matrix used in the CRRT system). The blood was inoculated with SUL and DUR as described above, and the chemostat jar was placed in a water bath at 37°C. Blood samples were collected at 0, 10, 20, 30, and 60 min and processed similar to the CRRT experiments described above.

Degradation was calculated as:


$$\%degradation=\frac{\left[abx\;0\right]-\left[abx\;x\right]}{\left[abx\;0\right]}*100,$$

where *abx* 0 is the concentration of SUL or DUR in the plasma at time zero, and *abx x* is the concentration of each drug in the control plasma at time *x* (e.g., 60 min). Degradation of less than 20% was considered negligible.

### Adsorption studies

Adsorption studies were conducted to evaluate if SUL or DUR sequester to either hemofilter set (M100 or HF1400). The CRRT model was modified to create a closed-circuit system where the effluent was rerouted to the central blood reservoir. Tap water was exogenously pumped into the effluent bag via a Masterflex Peristaltic pump (Cole-Parmer, Vernon Hills, IL, USA) at the same rate to prevent CRRT system abortion due to weight imbalance. At the same drug injection, bovine blood was also supplemented with urea (Sigma-Aldrich, St Louis, MO, USA) to reach a final concentration of approximately 75 mg/dL and serve as a control solute to account for drug dilution because it is known to not bind to the CRRT system. Blood urea nitrogen (BUN) was measured at the Hartford Hospital chemistry laboratory using a UREAL reagent assay on a COBAS 8000 analyzer (Roche Diagnostic, Indianapolis, IN) and multiplied by 2.14 to determine the endogenous urea content.

Blood samples were drawn from the central reservoir at 0, 10, 20, 30, and 60 min, processed as described above and then frozen immediately at −80°C until analysis. A total of 12 adsorption experiments were performed incorporating various CRRT modes (CVVH and CVVHD) and filters (M100 and HF1400) at a constant flow rate of 2 L/h.

Adsorption was calculated as the difference between the total amount of the drug added to the system and the total amount recovered in the effluent and blood at each measured time point over 60 min. Dilution and degradation were incorporated into the adsorption calculation as follows at each time point:


$$\%\;dilution\;factor=\frac{\left[urea\;0\right]-\left[urea\;x\right]}{\left[urea\;0\right]}*100,$$

where urea 0 is urea concentration in the central blood compartment at time 0, and urea *x* is urea concentration in the blood at time *x*.


$$\%\;adsorption=\frac{\left[C_{abx\;0}]-[C_{abx\;x}\right]}{\left[C_{abx\;0}\right]}*\;100-dilution\;factor\;(\%)-degradation\;(\%),$$

where *C_abx_*_0_ is concentration of SUL or DUR in the pre-filter plasma at time zero, and *C_abx x_* is the concentration of each agent in the pre-filter plasma at time *x* (e.g., 60 min). Adsorption of less than 20% was considered negligible.

### Bioanalytical procedures

SUL and DUR concentrations in bovine plasma, effluent fluid, and protein-free filtrate were assayed by a qualified non-GLP bioanalytical method using protein precipitation and liquid chromatography tandem mass spectrometry assay (LC-MS/MS) at Entasis (supplemental material).

### SUL and DUR CL_TM_

The sieving coefficient (SC, during CVVH) and saturation coefficient (SA, during CVVHD) of SUL and DUR were calculated as follows:


$$\begin{array}{r} SC=\frac{2*C_{uf}}{C_{pre}+C_{post}}\\ SA=\frac{2*C_{dialysate}}{C_{pre}+C_{post}} \end{array},$$

where *C*_uf_ is the concentration in the ultrafiltrate, *C*_pre_ is the concentration from the pre-filter sampling port, *C*_dialysate_ is the concentration in the dialysate, and *C*_post_ is the concentration from the post-filter sampling port. The CL_TM_ was then calculated for CVVH and CVVHD modes as follows:


$$\begin{array}{r} CL_{SC}\;(CVVH)=\frac{SC*Q_{uf}*Q_{b}}{Q_{b}+Q_{rep}}\\ CL_{SA}\;(CVVHD)=SA*Q_{d} \end{array},$$

where *Q*_uf_ is the ultrafiltration flow rate, *Q*_b_ is the blood flow rate, *Q*_rep_ is the pre-replacement fluid rate, and *Q*_d_ is the dialysate flow rate.

### Statistical analyses

A Student’s *t*-test was performed to assess the SC/SA differences between filters (M100 and HF1400), CRRT mode (CVVH versus CVVHD), and point of dilution in CVVH mode (100% pre- vs 50%/50% pre-/post-). Multiple linear regression was used to determine the influence of effluent flow rate, hemofilter, CRRT mode, and replacement fluid on SUL and DUR CL_TM_. All statistical analyses were conducted in SigmaPlot Version 14 (Systat Software, San Jose, CA). The effluent rate during CVVH was calculated as the sum of the total ultrafiltration rate (i.e., pre-blood pump fluid rate + pre-filter replacement fluid rate + post-filter replacement fluid rate + fluid removal rate), while the effluent rate during CVVHD was the sum of dialysate flow rate + fluid removal rate.

### Monte Carlo simulations and dose optimization

The goal of the simulations was to evaluate the PTA for pharmacodynamic targets and the achievable AUC exposures following dosing regimens included in the SUL-DUR drug label following different effluent dosages. Optimized dosing regimens were selected to achieve (i) a PTA > 90% for exposures that exceed what is needed to achieve 1-log of kill per the established pharmacodynamic targets for both SUL (≥50% *f*T>MIC) and DUR (*f*AUC/MIC ≥10) at the susceptibility breakpoint of 4/4 µg/mL, and then (ii) mean 24 h Day 2 total drug AUCs within ± one standard deviation for AUC exposures in patients treated during Phase 2 and Phase 3 clinical trial programs when corrected for CL_CR_. The detailed methodology and assumptions for these Monte Carlo simulations can be found in the supplemental material. A set of a priori criteria were used during the dosing regimen selection process. First, higher mean AUCs were weighted over lower mean AUCs if multiple dosing regimens resulted in values within the ± one standard deviation range. Second, SUL PTA and AUC were weighted over DUR (i.e., it was considered more important to have higher exposure of the active antibiotic component compared with the beta-lactamase inhibitor) provided both drugs achieved their established pharmacodynamic targets. Finally, the convenience of selecting dosing regimens already reported in the package insert was considered for simplicity.

## SUPPLEMENTAL MATERIAL

Supplemental material aac.01674-23-s0001.docx Tables S1 to S12, Fig. S1, and supplemental methods.

ASM does not own the copyrights to Supplemental Material that may be linked to, or accessed through, an article. The authors have granted ASM a non-exclusive, world-wide license to publish the Supplemental Material files. Please contact the corresponding author directly for reuse.
