# Supplementary material for: Ex vivo assessment of sulbactam-durlobactam clearance during continuous renal replacement therapy to guide dosing recommendations
Source: Antimicrob Agents Chemother. 2024 Dec 10;69(1):e01674-23. doi: 10.1128/aac.01674-23 (PMC11784458; doi:10.1128/aac.01674-23)
Supplement: Supplemental material — Tables S1 to S12, Fig. S1, and supplemental methods. [file aac.01674-23-s0001.docx]

Supplemental Data – November 11, 2024

***Ex Vivo* Assessment of Sulbactam-Durlobactam Clearance during Continuous Renal Replacement Therapy to Guide Dosing Recommendations**

Yasmeen Abouelhassan^1*^, Yuwei Shen^1^, April Chen^2**^, Xiaoyi Ye^3^,

David P. Nicolau^1, 4^, Joseph L. Kuti^1,#^

^1^Center for Anti-Infective Research and Development, Hartford Hospital, Hartford, CT, USA; ^2^Innoviva Specialty Therapeutics Inc., Waltham, MA, USA; ^3^Division of Nephrology, Hartford Hospital, Hartford, CT, USA; ^4^Division of Infectious Diseases, Hartford Hospital, Hartford, CT, USA

* Present affiliation: Merck & Co Inc, Rahway, NJ, USA

** Present affiliation: Astra Zeneca, Waltham, MA, USA

**Results**

## Table S1. Sulbactam (SUL) and durlobactam (DUR) adsorption to hemofilter set across different CRRT modes and hemofilters

|  |  | Average Adsorption over 60 minutes (%) | |
| --- | --- | --- | --- |
| CRRT mode | Filter | SUL | DUR |
| CVVH | M100 | 6.80 ± 5.52 | 7.53 ± 5.08 |
| CVVH | HF1400 | 8.36 ± 3.31 | 9.54 ± 3.12 |
| CVVHD | M100 | 11.15 ± 2.41 | 12.31 ± 3.08 |
| CVVHD | HF1400 | 10.81 ± 2.32 | 10.34 ± 2.71 |

## Figure S1. Final linear regression analysis of the CL_TM_ (L/h) of sulbactam (a) and durlobactam (b) based on the effluent flow rate (L/h) only

a) Sulbactam, R^2^=0.834, CL_TM_=0.114+(0.97*Effluent Rate)

b) Durlobactam, R^2^=0.84, CL_TM_=0.134+(0.76*Effluent Rate)

**Table S2**. Multiple linear regression analyses of the effect of filter type, replacement fluid, and effluent flow rate on SUL and DUR transmembrane clearance (CL_TM_) in CVVH mode only

|  | Coefficient | Std. Error | P-value |
| --- | --- | --- | --- |
| SUL (adjusted R^2^ = 0.949) |  |  |  |
| Constant | 0.086 | 0.105 | 0.421 |
| Filter type (1=M100, 0=HF1400) | -0.179 | 0.050 | <0.001 |
| Replacement fluid^a^ | 0.191 | 0.084 | 0.028 |
| Effluent flow rate^b^ | 0.863 | 0.032 | <0.001 |
| DUR (adjusted R^2^ = 0.917) |  |  |  |
| Constant | 0.140 | 0.108 | 0.205 |
| Filter type (1=M100, 0=HF1400) | 0.076 | 0.052 | 0.151 |
| Replacement fluid^a^ | -0.017 | 0.086 | 0.845 |
| Effluent flow rate^b^ | 0.690 | 0.033 | <0.001 |

Note. SUL, sulbactam; DUR, durlobactam; Std. Error, standard error; CRRT, continuous renal replacement

^a^ 1 = 100/0% pre-replacement, 0 = 50/50% pre-replacement

^b^ Effluent flow rates were assessed as continuous values in L/h.

**Table S3.** Sulbactam transmembrane clearance (CL_TM_) calculated from effluent flow rates based on the effluent rate (ER) model only compared with the model including mode (CVVH, CVVHD) and filter (M100, HF1400)

| Effluent flow rate (L/h) | CL_TM_ (L/h) | | | | |
| --- | --- | --- | --- | --- | --- |
|  | ER only | ER +  CVVH +  M100 | ER +  CVVH + HF1400 | ER +  CVVHD + M100 | ER +  CVVHD + HF1400 |
| 1 | 1.09 | 0.84 | 1.01 | 1.37 | 1.54 |
| 2 | 2.06 | 1.81 | 1.98 | 2.34 | 2.51 |
| 2.5 | 2.55 | 2.30 | 2.47 | 2.83 | 3.00 |
| 3 | 3.03 | 2.78 | 2.95 | 3.32 | 3.49 |
| 4 | 4.01 | 3.76 | 3.93 | 4.29 | 4.46 |
| 5 | 4.98 | 4.73 | 4.90 | 5.26 | 5.43 |

## Table S4. Durlobactam transmembrane clearance (CL_TM_) calculated from effluent flow rates based on the effluent rate (ER) model only compared with the model including mode (CVVH, CVVHD)

| Effluent flow rate (L/h) | CL_TM_ (L/h) | | |
| --- | --- | --- | --- |
|  | ER only | ER + CVVH | ER + CVVHD |
| 1 | 0.89 | 0.80 | 1.17 |
| 2 | 1.65 | 1.56 | 1.93 |
| 2.5 | 2.03 | 1.94 | 2.31 |
| 3 | 2.41 | 2.32 | 2.69 |
| 4 | 3.17 | 3.08 | 3.45 |
| 5 | 3.93 | 3.84 | 4.21 |

##

## Table S5. Probability of attaining 1-log kill target of sulbactam dosing regimens at different CRRT effluent flow rates compared with the population of patients with VABP not supported on CRRT with CRCL 30-129 ml/min

| CRRT Effluent flow rate (L/h) | Regimen (3h infusion) | PTA^a^ | | | | | Total AUC_24-48_ | | | | |
| --- | --- | --- | --- | --- | --- | --- | --- | --- | --- | --- | --- |
|  |  | 2 | 4 | 8 | 16 | 32 | mean | SD | CV | 25^th^ percentile | 75^th^ percentile |
| CRCL 30-129 ml/min | 1g q6h | 1 | 1 | 0.14 | 0 | 0 | 466 | 109 | 23 | 376 | 542 |
| 1 | 1g q8h | 1 | 1 | 1 | 0.21 | 0 | 533 | 108 | 20 | 451 | 598 |
| 1 | 1g q12h^b^ | 1 | 1 | 0.53 | 0 | 0 | 362 | 81 | 22 | 301 | 408 |
| 2 | 1g q6h | 1 | 1 | 1 | 0.41 | 0 | 607 | 112 | 19 | 523 | 671 |
| 2 | 1g q8h | 1 | 1 | 1 | 0.04 | 0 | 456 | 82 | 18 | 395 | 503 |
| 2.5 | 1g q6h | 1 | 1 | 1 | 0.271 | 0 | 563 | 98 | 17 | 488 | 620 |
| 2.5 | 1g q8h | 1 | 1 | 0.98 | 0.01 | 0 | 420 | 73 | 17 | 366 | 461 |
| 3 | 1g q6h | 1 | 1 | 1 | 0.16 | 0 | 526 | 85 | 16 | 459 | 580 |
| 3 | 1g q8h | 1 | 1 | 0.93 | 0 | 0 | 399 | 66 | 16 | 350 | 440 |
| 4 | 1g q6h | 1 | 1 | 1 | 0.02 | 0 | 469 | 64 | 14 | 418 | 514 |
| 4 | 1g q8h | 1 | 1 | 0.71 | 0 | 0 | 348 | 50 | 14 | 310 | 378 |
| 5 | 1g q6h | 1 | 1 | 1 | 0 | 0 | 420 | 53 | 13 | 378 | 453 |
| 5 | 1g q4h | 1 | 1 | 1 | 0.54 | 0 | 622 | 78 | 12 | 562 | 671 |

CRRT, continuous renal replacement therapy; VABP, ventilator associated bacterial pneumonia; CRCL: creatinine clearance; PTA, probability of target attainment; AUC, area under the curve from 24-48 hours; SD, standard deviation; CV, coefficient of variability

^a^ 1-log kill target for sulbactam was 50% *f*T>MIC

^b^ q12h regimen simulated for 48-72h; all other regimens are 24-48h

## Table S6. Probability of attaining 1-log kill target of durlobactam dosing regimens at different CRRT effluent flow rates compared with the population of patients with VABP not supported on CRRT with CRCL 30-129 ml/min

| CRRT Effluent flow rate (L/h) | Regimen (3h infusion) | PTA^a^ | | | | | Total AUC_24-48_ | | | | |
| --- | --- | --- | --- | --- | --- | --- | --- | --- | --- | --- | --- |
|  |  | 2 | 4 | 8 | 16 | 32 | mean | SD | CV | 25^th^ percentile | 75^th^ percentile |
| CRCL 30-129 ml/min | 1g q6h | 1 | 1 | 1 | 1 | 0.97 | 452 | 68 | 15 | 399 | 491 |
| 1 | 1g q8h | 1 | 1 | 1 | 1 | 1 | 735 | 144 | 20 | 629 | 812 |
| 1 | 1 g q12h^b^ | 1 | 1 | 1 | 1 | 0.98 | 505 | 107 | 21 | 427 | 564 |
| 2 | 1g q6h | 1 | 1 | 1 | 1 | 1 | 838 | 146 | 17 | 729 | 926 |
| 2 | 1g q8h | 1 | 1 | 1 | 1 | 1 | 630 | 109 | 17 | 549 | 696 |
| 2.5 | 1g q6h | 1 | 1 | 1 | 1 | 1 | 777 | 126 | 16 | 684 | 854 |
| 2.5 | 1g q8h | 1 | 1 | 1 | 1 | 1 | 581 | 92 | 16 | 513 | 638 |
| 3 | 1g q6h | 1 | 1 | 1 | 1 | 1 | 726 | 113 | 16 | 639 | 789 |
| 3 | 1g q8h | 1 | 1 | 1 | 1 | 1 | 544 | 84 | 15 | 482 | 595 |
| 4 | 1g q6h | 1 | 1 | 1 | 1 | 1 | 636 | 88 | 14 | 571 | 690 |
| 4 | 1g q8h | 1 | 1 | 1 | 1 | 1 | 478 | 64 | 13 | 431 | 523 |
| 5 | 1g q6h | 1 | 1 | 1 | 1 | 1 | 566 | 72 | 13 | 510 | 609 |
| 5 | 1g q4h | 1 | 1 | 1 | 1 | 1 | 844 | 101 | 12 | 769 | 906 |

CRRT, continuous renal replacement therapy; VABP, ventilator associated bacterial pneumonia; CRCL: creatinine clearance; PTA, probability of target attainment; AUC, area under the curve from 24-48 hours; SD, standard deviation; CV, coefficient of variability

^a^ 1-log kill target for durlobactam was *f*AUC/MIC ≥ 10

^b^ q12h regimen simulated for 48-72h; all other regimens are 24-48h

## Table S7. Sensitivity analyses determining probability of attaining 1-log kill target of sulbactam dosing regimens using transmembrane clearance (CL_TM_) estimated multivariate linear regression including CVVH+AN69 (lowest CL_TM_) versus CVVHD+HF1400 (highest CL_TM_)

| CRRT Effluent flow rate (L/h) | Regimen (3h infusion) | CL_TM_ Model | PTA^a^ | | | | | Total AUC_24-48_ | | | | |
| --- | --- | --- | --- | --- | --- | --- | --- | --- | --- | --- | --- | --- |
|  |  |  | 2 | 4 | 8 | 16 | 32 | mean | SD | CV | 25^th^ percentile | 75^th^ percentile |
| 2 | 1g q8h | CVVH+AN69 | 1 | 1 | 1 | 0.06 | 0 | 469 | 89 | 19 | 403 | 516 |
| 2 | 1g q8h | CVVHD+HF1400 | 1 | 1 | 0.99 | 0.01 | 0 | 423 | 73 | 17 | 368 | 466 |
| 3 | 1g q6h | CVVH+AN69 | 1 | 1 | 1 | 0.22 | 0 | 546 | 89 | 16 | 476 | 602 |
| 3 | 1g q6h | CVVHD+HF1400 | 1 | 1 | 1 | 0.10 | 0 | 499 | 76 | 15 | 440 | 549 |
| 3 | 1g q8h | CVVH+AN69 | 1 | 1 | 0.96 | 0 | 0 | 409 | 68 | 17 | 356 | 447 |
| 3 | 1g q8h | CVVHD+HF1400 | 1 | 1 | 0.83 | 0 | 0 | 373 | 58 | 16 | 329 | 406 |

^a^ 1-log kill target for sulbactam was 50% *f*T>MIC

## Table S8. Sensitivity analyses determining probability of attaining 1-log kill target of durlobactam dosing regimens using transmembrane clearance (CL_TM_) estimated multivariate linear regression including CVVH (lowest CL_TM_) versus CVVHD (highest CL_TM_)

| CRRT Effluent flow rate (L/h) | Regimen (3h infusion) | CL_TM_ Model | PTA^a^ | | | | | Total AUC_24-48_ | | | | |
| --- | --- | --- | --- | --- | --- | --- | --- | --- | --- | --- | --- | --- |
|  |  |  | 2 | 4 | 8 | 16 | 32 | mean | SD | CV | 25^th^ percentile | 75^th^ percentile |
| 2 | 1g q8h | CVVH | 1 | 1 | 1 | 1 | 1 | 645 | 110 | 17 | 560 | 710 |
| 2 | 1g q8h | CVVHD | 1 | 1 | 1 | 1 | 1 | 595 | 95 | 16 | 526 | 651 |
| 3 | 1g q6h | CVVH | 1 | 1 | 1 | 1 | 1 | 735 | 117 | 16 | 645 | 802 |
| 3 | 1g q6h | CVVHD | 1 | 1 | 1 | 1 | 1 | 687 | 100 | 15 | 611 | 752 |
| 3 | 1g q8h | CVVH | 1 | 1 | 1 | 1 | 1 | 556 | 86 | 15 | 491 | 610 |
| 3 | 1g q8h | CVVHD | 1 | 1 | 1 | 1 | 1 | 512 | 73 | 14 | 458 | 559 |

^a^ 1-log kill target for durlobactam was *f*AUC/MIC ≥ 10

## Materials and Methods

## *Protein binding.* The purpose of these studies was to assess SUL and DUR protein binding in the bovine blood. Approximately 0.9 mL plasma samples (without the protease inhibitor) obtained from the central blood reservoir at 0 min and from the pre-filter blood at 60 min were aliquoted to Centrifree® Ultrafiltration Device (Merck Millipore Ltd, Tullagreen, Carrigtwohill, County Cork, Ireland) and centrifuged in a fixed-angle rotor at 1500 xg, 4°C, 45 minutes to generate the protein free fraction (PFF). The resulting PFF and the aliquots of plasma samples were stored at -80°C until analysis for SUL and DUR concentrations in each matrix.

$$\% protein binding=100-\frac{concentration in PFF}{Concentration in plasma} * 100$$

## *Bioanalytical procedures.* SUL and DUR concentrations in bovine plasma, effluent fluid, and PFF were assayed by a qualified non-GLP bioanalytical method using protein precipitation and liquid chromatography tandem mass spectrometry assay (LC-MS/MS) at Innoviva Specialty Therapeutics Inc (Waltham, MA). The LC-MS/MS method is summarized in Table S9. Calibration standards and Quality Control (QC) were prepared in blank bovine plasma supplemented with equal volume of SigmaFast solution by serial dilution to quantify the plasma sample concentrations. The SigmaFast solution (Sigma Aldrich part #: S8820) was made fresh on the day of analysis by dissolving 1 tablet of SigmaFast in 10mL of DI water. A dilution factor of 2 was applied to quantify the unknown plasma sample concentrations to account for the dilution with equal volume of SigmaFast solution. Calibration standards and Quality Control (QC) were prepared in 0.9% normal saline by serial dilution to quantify the effluent samples and PFF samples. 25 µL of calibration standards and QC along with unknown samples were aliquoted in 96-well plates followed by protein precipitation with 300 µL of crash solution (100% acetonitrile containing 0.1% formic acid and 250 ng/mL of carbutamide as internal standard). Vortex and centrifuge the plate and the supernatant was transferred for LC-MS/MS analysis. The analytical specifications are summarized in Table S10.

## Table S9. LC-MS/MS Conditions for SUL and DUR Bioanalysis

| Instrument | Schimadzu UPLC - Sciex QTrap6500 LC/MS/MS Mass Spectrometer | | |
| --- | --- | --- | --- |
| Column | Atlantis T3, 5µ, 50 x 3.0mm | | |
| Column Temperature | 35^0^C | | |
| Sample Temparature | 10^0^C | | |
| Flow rate | 1.200 mL/min | | |
| Gradient | Time (min) | %B | Curve |
|  | Initial | 2.0 |  |
|  | 0.3 | 2.0 | 6 |
|  | 1.3 | 98 | 6 |
|  | 1.75 | 98 | 6 |
|  | 1.76 | 2.0 | 6 |
|  | 2.00 | Stop |  |
|  |  |  |  |
| Divert Valve | 0.30 min to Mass Spec  1.80 min to waste | | |
| Mobile Phase A | 0.1% formic acid in water | | |
| Mobile Phase B | 0.1% formic acid in Acetonitrile | | |
| MRM | Source Type Turbo Spray  Polarity: Negative  Resolution Q1: Unit  Resolution Q3: Unit  IS: -3500.00  TEM: 550.00  CUR: 30.00  GS1: 70.00  GS2: 60.00  CAD: Medium | | |
| Injection volume | 0.5 µL | | |

**MRM Transitions**:

| Compound ID | Q1 | Q3 | DP | CE | CXP |
| --- | --- | --- | --- | --- | --- |
| SUL | 231.9 | 139.9 | -5 | -18 | -21 |
| DUR | 275.9 | 96.9 | -40 | -26 | -13 |
| Carbutamide (IS) | 270.0 | 171.0 | -55 | -25 | -10 |

## Table S10. Analytical Method Specifications

| **Analyte** | **Matrix** | **LLOQ (µg/mL)** | **ULOQ (µg/mL)** |
| --- | --- | --- | --- |
| SUL | Bovine plasma | 0.005 | 50 |
| DUR | Bovine plasma | 0.005 | 50 |
| SUL | 0.9% Normal saline | 0.005 | 50 |
| DUR | 0.9% Normal saline | 0.005 | 50 |
| LLOQ, lower limit of quantification; ULOQ, upper limit of quantification. Note. Upon linear regression of the peak area ratios of SUL/IS and DUR/IS, fortified calibration standards and QC samples were with ±20% of target suggesting the analytical run supporting SUL and DUR concentrations in the unknown samples was valid. | | | |

***Monte Carlo simulations.*** To obtain the standard deviation ranges for VABP patients with normal kidney function receiving the standard dose, a 1000 patient Monte Carlo simulation using the non-parametric simulator in Pmetrics for R was performed using parameters obtained from the two compartment population pharmacokinetic analysis of 162 patients from phase 2 and phase 3 clinical trials. The simulated dose included the approved standard dosing regimen (1g-1g every 6h as 3h infusion) for patients with a mean CL_CR_ of 80 ml/min (range: 30-129 mL/min). After 8 simulated doses of 1g-1g q6h as 3h infusions, the total drug AUC_24-48_ were calculated by trapezoidal rule for each simulated patient and compared with the Day 2 AUCs in patients with CL_CR_ of 30-129 ml/min for use as a target reference for CRRT total drug AUC_24-48_ exposures. The parameter estimates used during Monte Carlo simulation of the VABP population are provided in Table S11.

For simulations at different effluent flow rates, renal clearance (CL_R_) from the population PK model was substituted with the CL_TM_ obtained from the *ex vivo* studies. SUL and DUR non-renal clearance (CL_NR_) (derived from patients with chronic renal insufficiency in Phase 1 studies) were 4.79 and 3.18 L/h, respectively (data on file, Innoviva Specialty Therapeutics Inc). A 30% coefficient of variability (CV) was added to the CL_NR_ values to account for patient variability because the non-renal clearance of these drugs in acute kidney injury is currently unknown. A 12% CV was added to the CL_TM_ values across all simulated effluent flow rates, as this was the average variability seen across the ex vivo studies. The simulated parameters and their dispersion are reported in Table S12. The dose of SUL-DUR 1g-1g every 6 hours as a 3h infusion was simulated across fixed effluent rates of 1, 2, 2.5, 3, 4, and 5 L/h. Additional dosing regimens including 1g-1g every 4, 8, and 12 hours (all as 3h infusions) were simulated across select effluent rates based on the exposure results from the standard dose at that rate. The protein binding of SUL and DUR was fixed at 38% and 10%, respectively, during all simulations.

Additional sensitivity analyses (Tables S7 and S8) were conducted by simulating different mode and filter scenarios (e.g., CVVH+M100 vs CVVHD+HF1400) as these variables were also significant in the initial multilinear regression models.

## **Table S11.** Pharmacokinetic parameters and dispersion used for sulbactam (SUL) and durlobactam (DUR) during the Monte Carlo simulations of VABP patients receiving standard 1g-1g q6h, 3h infusion dosage

|  | SUL | DUR |
| --- | --- | --- |
| CL_CR_ (ml/min) | 80 ml/min (range: 30-129 ml/min) | |
| Wt (kg) | 76.4 ±4.7 | |
| Total Body CL (L/h)^a^ | (4.79+(0.648*13.6*((CL_CR_/ 90)^0.932)))*((Wt/75)^1.03)*(1+ CLINF) | (3.18+(0.66*9.36*((CL_CR_/ 90)^0.694))) *((Wt/75)^0.664) |
| V_c_ (L)^b^ | 12.1*(wt/75)^0.828)*(1+VcINF) | 12.5*(wt/75)^0.518)*(1+VcINF)) |
| Q (L/h) | 7.82 | 4.42 |
| V_p_ (L) | 6.98 ± 6.66 | 5.83 ± 5.27 |

CL_CR_, creatinine clearance; Wt, weight; CL, clearance; V_c_, volume of central compartment; Q, intercompartmental transfer constant; V_p_, volume of peripheral compartment

^a^ CLINF is the Theta constant describing impact of infection type on clearance. For SUL, -0.294 was used to simulate VABP patients.

^b^ VcINF is the Theta constant describing impact of infection type on Vc. For SUL, 1.38 was used to simulate HABP/VABP patients. For DUR, 1.52 was used to simulate HABP/VABP patients.

## **Table S12.** Pharmacokinetic parameters, dispersion, and ragnes used for sulbactam (SUL) and durlobactam (DUR) during the Monte Carlo simulations of varying CRRT effluent flow rates

|  | SUL | DUR |
| --- | --- | --- |
| CL_NR_ (L/h) | 4.79 ± 1.44 (range: 2-7) | 3.18 ± 0.95 (range: 1.3-5) |
| CL_R_ (L/h) | see Table S3 | see Table S4 |
| Wt (kg) | 76.4 ± 4.7 | 76.4 ± 4.7 |
| Total Body CL (L/h) | CL_NR_ + CL_R_ | CL_NR_ + CL_R_ |
| V_c_ (L) | 12.1*(wt/75)^0.828)*(1+1.38) (range: 20-45) | 12.5*(wt/75)^0.518)*(1 + 1.52)) (range: 16-38) |
| Q (L/h) | 7.82 (range: 7-8) | 4.42 (range: 4-5) |
| V_p_ (L) | 6.98 ± 6.66 (range: 1-30) | 5.83 ± 5.27 (range: 1-16) |

CL_NR_, non-renal clearance; CL_R_, renal clearance; Wt, weight; CL, clearance; V_c_, volume of central compartment; Q, intercompartmental transfer constant; V_p_, volume of peripheral compartment
